# Underlying mechanisms of phosphodiesterase 10A and glutamate-ammonia ligase genes that regulate inosine monophosphate deposition and thereby affect muscle tenderness in Jingyuan chickens

**DOI:** 10.5713/ab.21.0134

**Published:** 2021-09-15

**Authors:** Weizhen Wang, Juan Zhang, Honghong Hu, Baojun Yu, Jintong He, Tingting Yao, Yaling Gu, Zhengyun Cai, Guosheng Xin

**Affiliations:** 1 Agriculture School, Ningxia University, Ningxia, 750021, China; 2 Ningxia Feed Engineering Technology Research Center, School of Life Sciences, Ningxia University, Ningxia, 750021, China

**Keywords:** Glutamate-ammonia Ligase (GLUL), Inosine Monophosphate, Jingyuan Chicken, Phosphodiesterase 10A (PDE10A), RNA-seq

## Abstract

**Objective:**

Inosine monophosphate (IMP) is a key factor that imparts of meat flavor. Differences in the IMP content in the muscles were evaluated to improve chicken meat quality.

**Methods:**

For this study, the IMP content was detected by high performance liquid chromatography. The gene expression profiles of Jingyuan chickens with different feeding patterns and different sexes were analyzed by RNA-sequencing (RNA-seq).

**Results:**

Breast muscle IMP content in free-range chickens was extremely significantly higher than that of caged chickens (p<0.01). Breast muscle IMP content in hens was also higher than that of cocks, but the difference was not significant. Correlation analysis showed that the breast muscle IMP content in caged hens and cocks was negatively correlated with the shear force, and the breast muscle IMP content in free-range hens was significantly negatively correlated with the shear force (p<0.05). The two key genes associated with IMP synthesis in chickens with different feeding patterns were glutamate-ammonia ligase (*GLUL*) and phosphodiesterase 10A (*PDE10A*). Bioinformatics analysis revealed that the *GLUL* and *PDE10A* genes are involved in glutamine biosynthesis and purine salvage pathways respectively. In addition, GLUL expression was positively correlated with the IMP content in caged and free-range chickens, and PDE10A expression was significantly positively correlated with the IMP content in caged and free-range chickens (p<0.05).

**Conclusion:**

These findings will facilitate the comprehension of the deposition of IMP in the muscles and thereby aid the process of selection and breeding of good quality local chickens.

## INTRODUCTION

Over the past few decades, worldwide there has been a marked increase in the consumption of poultry meat and eggs [1,2]. There are various reasons for this increased growth, such as genetic selection, nutritional levels, feed formulation and production technology. However, the main reason for the increased demand for poultry meat and eggs is that it is a short-cycle, cheap source of animal protein [3,4]. This increasing consumer demand has led researchers to focus more on poultry feed remuneration, growth rates, and muscle yields, resulting in a steady decrease in the quality of the poultry meat [5]. Along with the continued prosperity of China’s economy, there is a pursuit of high quality of life and the decline in the quality of poultry meat has become a contradiction faced by consumers. Therefore, ensuring the same supply capacity while improving the quality of poultry meat is essential for the farmers to resolve this conflict.

The higher the inosine monophosphate (IMP) content in the muscles, the better the flavor and texture of the meat [6]. Studies have shown that feeding pattern and sex of the chicken have an impact on the IMP content in the muscles [7–9]. Detection of endogenous compounds in five local chicken species in Korea revealed higher levels of IMP in the breast and leg muscles of hens than those in cocks, while the levels of inosine in the breast and leg muscles were remained unaffected by sex [7]. A study on caged and free-range chickens revealed that the IMP content in the breast and leg muscles of free-range Lueyang black-bone chicken, Beijing You chicken, and Guangxi Yellow chicken was significantly higher than that in the caged chickens [8–10]. In male and female Lueyang black-bone chickens, the IMP content in hens was significantly higher than that of cocks [11]. Moreover, the IMP content was significantly and positively correlated with the expressions of phosphoribosyl pyrophosphate amidotransferase (PPAT), glycinamide ribonucleotide synthetase-aminoimidazole ribonucleotide synthetase-glycinamide ribonucleotide transformylase (GARS-AIRS-GART), adenosine monophosphate deaminase 1 (AMPD1), and guanosine monophosphate reductase (GMPR) [11]. Transcriptome sequencing of Lueyang black-bone chickens revealed that the expressions of AMPD1, 5′-nucleotidase, cytosolic IA (NT5C1A), and ectonucleoside triphosphate diphosphohydrolase 8 (ENTPD8) in 60-day-old free-range chickens and GART, GARS, and adenylosuccinate lyase (ADSL) in 120-day-old free-range chickens were significantly higher than that of caged chickens of the same age [8].

Jingyuan chicken, a local breed that was domesticated by farmers in the northern mountainous area of Pengyang County, Ningxia in a long-term domestication process of free-range breeding, is one of the protected species of Chinese livestock and poultry resources. There are three core groups of white, hemp and black feathers, which are widely distributed in the Loess Plateau region of southern Ningxia. The cocks are medium-sized, with a broad chest and back and sturdy tibiae, while the hens have a broad back and a round abdomen; adult cockerels weigh 1,888 to 2,250 g and hens weigh 1,630 to 1,670 g [6]. Sexual maturity is late in Jingyuan chickens, and the hens usually start laying eggs at 8 to 9 months of age with an annual egg production of 117 to 124 [6]. Due to the unique geographical environment, Jingyuan chicken meat is delicious and tender, with high amino acid and fatty acid content and low cholesterol levels; it is especially high in polyunsaturated fatty acids that have a special nutritional value for the human body. In this study, our aim was to explain the differences in the IMP content in the muscles with respect to different feeding patterns and different sexes of the chickens. We intended to study the changes at the level of gene transcription, based on physical indicators and breast muscle IMP content in Jingyuan chickens. Our goal was to screen for key candidate marker genes that regulate IMP deposition in the muscles and thus provide a scientific basis for the selection and breeding of high-quality local broiler chickens.

## MATERIALS AND METHODS

### Ethics statement

All experimental procedures were performed according to the Guide for Animal Care and Use of Laboratory Animals of the Institutional Animal Care and Use Committee of Ningxia University. The animal use protocol listed below has been reviewed and approved by the Animal Ethical and Welfare Committee (NXUC3127858).

### Experimental animals

The Jingyuan chickens used in this study were from the Zhaona Chicken Breeding Center in Pengyang County, Ningxia. All the chickens were grown under the same management conditions and given the same feeding. Thereafter, 45 chickens (15 males and 30 females) were slaughtered when they were 180 days old. Tissue samples were rapidly collected from the breast muscles of the slaughtered chickens; these tissues were immediately snap-frozen in liquid nitrogen tanks and stored in the laboratory in −80°C and −20°C refrigerators for subsequent experiments.

### Determination of shear force, water holding capacity, and cooking loss

Shear force was determined according to the Warner-Bratzler method [12]. For this purpose, the meat was placed in a constant temperature water bath at 80°C and when the temperature of the center of the meat sample reached 75°C, it was removed and cooled down to the room temperature. Samples of 1.0 cm×1.0 cm×3.0 cm were cut perpendicular to the direction of the muscle fibers with a sampler and the tenderness of these meat samples was measured using an RH-N50 tenderness tester (Tenovo, Beijing, China). The same process was repeated three times for each sample, and the average value was noted. The contents of water holding capacity (WHC) and cooking loss were determined using the method described by Dalgaard et al [12]. The WHC of the meat samples was measured by Bulader-M10 meat water loss tester (Bulader, Beijing, China). A meat sample of 1.0 cm in diameter and 1.0 cm thickness was excised from the breast muscle in the direction of the muscle fibers with a sharp circular sampler. The initial weight of the sample was marked as W1. Thereafter, it was placed between two layers of gauze with 20 layers of filter paper on top and bottom and pressurized to 35 kg for 5 min. The sample was again weighed, and this weight was marked as W2. The process was repeated three times for each sample was repeatedly measured three times and the average value was noted. The WHC of the meat samples was expressed as a measure of the water loss as follows: water loss (%) = (W1–W2)/W1×100. To determine the cooking loss, the breast muscle tissue was cut into 2.0 cm×2.0 cm×3.0 cm meat samples; the fat and connective tissues were removed from the surface of the muscle and the initial weight of the sample was marked as Z1. Thereafter, the tissue samples were placed in boiling water in an aluminum steamer for 30 min, kept in a cool ventilated place for 15 min, and again weighed; this weight was marked as Z2. The same process was repeated three times for each sample, and the average value was noted. The cooking loss was expressed as, cooking loss (%) = (Z1–Z2)/Z1×100.

### Determination of inosine monophosphate and inosine content

The contents of IMP and inosine were determined using the method described by Jung et al [7]. Breast muscle samples, 10 g, were ground in a meat grinder and each sample was divided into three portions weighing 2 g, accurate to 0.0001 g. The samples were placed in a 15 mL centrifuge tube with 5 mL of 6% perchloric acid. Subsequently, these samples were homogenized at high speed, followed by centrifugation of the homogenate at 8,000 rpm for 10 min; the centrifuged samples were filtered into a 100 mL volumetric flask. The filter residues were again homogenized with 5 mL of 6% perchloric acid, and the homogenate was centrifuged and filtered. The two supernatants were thus combined and adjusted to pH 6.5 with 5.0 mol/L and 0.5 mol/L NaOH, and then mixed well. This sample was then filtered through a filter screen, and the filtrate was collected for high-performance liquid chromatography analysis.

The chromatographic conditions were as follows: Waters Atlantis dC18 chromatographic column (5 μm, 4.6 mm×150.0 mm) was used and the column temperature was 25°C. The mobile phase consisted of a 15:85 mixture of liquid A and liquid B, where liquid A was methanol and liquid B was 0.05 mol/L of a 1:1 mixture of dipotassium hydrogen phosphate and potassium dihydrogen phosphate (liquid B was prepared on the spot, filtered by 0.45 μm membrane, and degassed by ultrasonication). The flow rate was 1 mL/min, injection volume was 10 μL, detection wavelength was 250 nm.

### Complementary DNA library construction and RNA sequencing

The Trizol method was used to extract the total RNA from nine breast muscle tissue samples excised from the Jingyuan chickens. The total RNA was tested for integrity by 1% gel electrophoresis, and the concentration and purity of the RNA samples were detected by Nanodrop ND-2000 (Therom, Waltham, MA, USA) and Agilent 2100 bioanalyzer (Agilent, Santa Clara, CA, USA). All the RNA samples with RNA integrity numbers >8.0 and absorbance 260:280 ratios >1.9 were selected for library construction and deep sequencing. A total amount of 10 μg RNA per sample was used as input material for the RNA sample preparations. Sequencing libraries were generated using NEB Next UltraTM RNA Library Prep Kit for Illumina (NEB, Beijing, China) and following the procedures and standards of the kit’s manual. For this process, mRNA was purified from the total RNA using Oligo (dt) magnetic beads, followed by fragmentation of the mRNA under elevated temperature in NEBNext First Strand Synthesis Reaction Buffer (5×). First strand cDNA was synthesized using a random hexamer primer and reverse transcriptase. Subsequently, second strand cDNA synthesis was performed using DNA polymerase I (DNA-pol I) and ribonuclease H (RNase H). After poly-A of the 3′ ends of the DNA fragments, NEBNext adapter with a hairpin loop structure was ligated to prepare the DNA for hybridization. AMPure XP beads (Beckman Coulter, Beverly, MA, USA) were used to select cDNAs around 350 to 400 bp in length. Subsequently, the second strand of U-containing cDNA was degraded using the USER enzyme (NEB, China), and finally the cDNA samples were amplified by polymerase chain reaction (PCR). The cDNA library quality was assessed with an Agilent 2100 bioanalyzer. Finally, 9 libraries were sequenced using an Illumina HiSeqTM2500 (Illumina, San Diego, CA, USA).

### RNA-seq data analysis

To get clean reads, the raw sequencing data were quality controlled using FastQC software, that is, clean reads were obtained after removing the reads containing adapters, low quality reads, and reads containing poly-N ≥10%. The Q20, Q30, and content of guanine and cytosine contents of the clean reads were calculated based on the abovementioned criteria. The clean reads that were obtained after the quality control process were mapped to the chicken reference genome via Hisat2 and Bowtie2 (ftp://ftp.ensembl.org/pub/current_fasta/gallus_gallus/dna/Gallus_gallus.Gallus_gallus-5.0.dna.toplevel.fa.gz). The gene expression level was estimated by the number of normalized fragments per kilobase of transcript per million fragments (FPKM) method. Differentially expressed genes (DEGs) among the samples were analyzed using the edgeR package. Genes that had an adjusted p-value <0.05 and a fold change (|log_2_FC|)>1 were considered as DEGs.

### Gene ontology and pathway analysis

The obtained DEGs required an insight into their biological functions to uncover important regulatory factors; this is usually analyzed using gene ontology (GO) (http://www.geneontology.org/) in bioinformatics analysis [13]. GO functional enrichment analysis can locate gene functions in three aspects: cellular components, biological processes, and molecular functions. Transcripts with similar annotations can be integrated and categorized using the GO annotation enrichment analysis. Biological pathway enrichment analysis relies heavily on the Kyoto encyclopedia of genes and genomes database (KEGG) ( http://www.genome.jp/kegg/) [13]. Pathway enrichment analysis can pinpoint the major metabolic transductions and signaling pathways of the DEGs. GO and KEGG enrichment analyses were performed using the GOseq based on Wallenius non-central hyper-geometric distribution and clusterprofiler R package, with a setting of p<0.05 as the significant enrichment threshold.

### Protein interaction network analysis

Protein interaction networks were analyzed by String protein interaction database ( http://string-db.org/) for proteins encoded by DEGs and edited visually using the Cytoscape v3.7.0 software [14].

### Reactome pathway and network cluster analysis

Gene set enrichment analysis, construction of molecular transformation networks, and metabolic mapping were carried out through the Reactome pathway database ( https://reactome.org/) [15]. Network cluster analysis was performed by the String protein interaction database.

### Quantitative real-time polymerase chain reaction verification

Based on the published gene sequences of ethanolamine-phosphate phospho-lyase (*ETNPPL*), acyl-CoA synthetase long-chain family member 4 (*ACSL4*), phosphodiesterase 10A (*PDE10A*), glutamate-ammonia ligase (*GLUL*), ATP synthase F1 subunit alpha W chromosome (*ATP5A1W*), ATP synthase F1 subunit alpha Z chromosome (*ATP5A1*), histidine triad nucleotide binding protein W (*HINTW*), and beta-actin (*β-actin*) on GeneBank, primers were designed using the primer 5.0 software (Table 1) and sent to the Sangon Company for synthesis. Total RNA was extracted from the pectoral muscle samples, using *β-actin* as the housekeeping gene. cDNA was synthesized using PrimeScript RT reagent Kit with gDNA Eraser (Takara, Dalian, China). Quantitative Real-time PCR (qRT-PCR) amplification was performed using SYBR premix Ex TaqTM II kit (Takara, China). Three replicates were performed for each sample. The relative gene expression levels were calculated using the 2^−ΔΔCt^ method [16].

### Data processing

One-way analysis of variance (ANOVA) was performed using IBM SPASS Statistics 23.0 software, and the results were expressed as mean±standard error; the correlation of IMP content with shear force and the relative gene expression was assessed using bivariate correlation analysis.

## RESULTS

### Shear force, water holding capacity, and cooking loss

Shear force, WHC, and cooking loss were calculated for the tissue samples excised from the breast muscles of Jingyuan chickens reared under the different feeding management conditions. A one-way ANOVA of these results showed that the breast muscle shear force in caged chickens was significantly higher than that in free-range chickens (p<0.05), while the cooking loss in meat of caged chickens was extremely significantly higher than that in the meat of free-range chickens (p<0.01). With respect to the different sex of the Jingyuan chickens, breast muscle shear force was extremely significantly higher in hens than in cocks (p<0.01), while WHC was significantly higher in hens than in cocks (p<0.05) (Table 2).

### Inosine monophosphate and inosine content

IMP and inosine content were measured in the tissue samples excised from the breast muscles of Jingyuan chickens reared under the different feeding management conditions. A one-way ANOVA of these results showed that the breast muscle IMP content in free-range chickens was extremely significantly higher than that of caged chickens (p<0.01), while the inosine content in caged chickens was extremely significantly higher than that of free-range chickens (p<0.01). Moreover, with respect to the different sex of the Jingyuan chickens, breast muscle IMP content in hens was higher than that in cocks, while the breast muscle inosine content in cocks was higher than that in hens, but neither of these were significantly different (Table 3).

### Inosine monophosphate content and shear force correlation analysis

**Figure 1:**
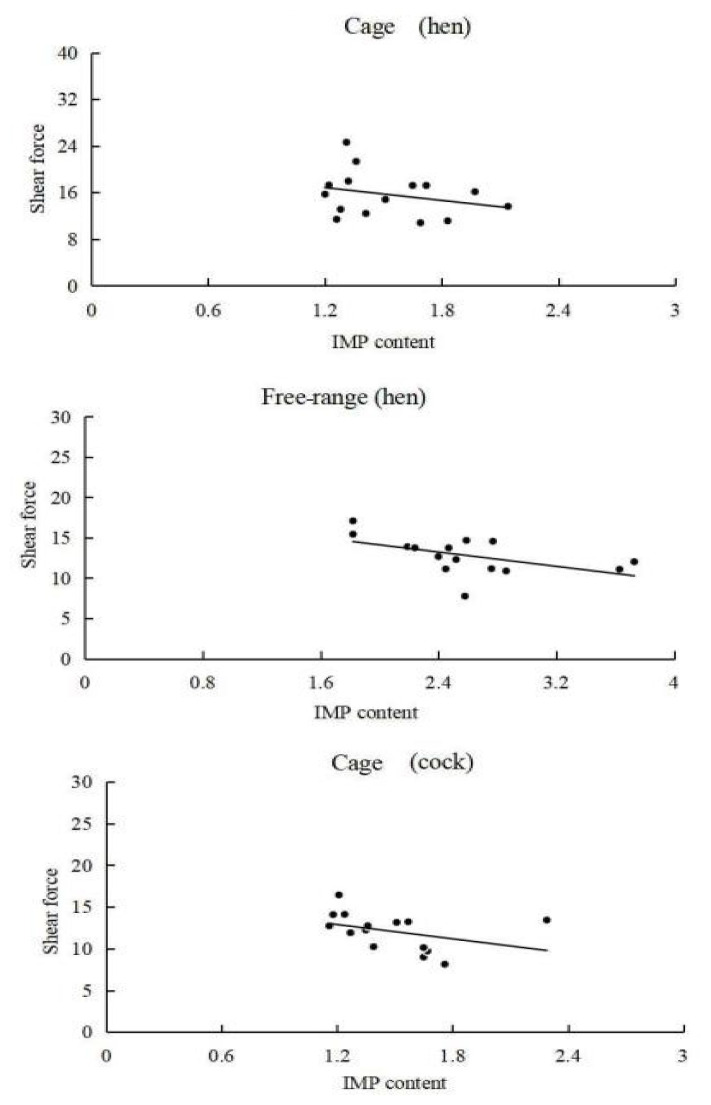
Scatter plot (regression line) of IMP content with shear force. IMP, inosine monophosphate.

Bivariate correlation analysis of the IMP content in the breast muscle tissue samples and the shear force of the breast muscles showed that the correlation in case of caged hens is R^2^ = −0.278 (p>0.05), in case of free-range hens it is R^2^ = −0.522 (p<0.05), and in case of caged cocks it is R^2^ = −0.38 (p>0.05). The scatter plot (regression line) of IMP content in breast muscle tissues with shear force of breast muscles visually reflects the relationship (Figure 1). The IMP content in the breast muscle tissues of free-range hens was significantly negatively correlated with the breast muscle shear force (p<0.05), and the breast muscle shear force was negatively correlated with the IMP content in breast muscles tissues of caged hens and cocks.

### RNA-seq quality control results

Sequencing of cDNA libraries from nine breast muscle tissue samples showed over 40.12 million raw reads per sample sequenced. After the quality control of the raw reads and the subsequent removal of the low quality reads, over 38.94 million clean reads were obtained per sample. The nucleobase content distribution showed that the GC content was stable between 55.86% and 56.72%, and the nucleobase composition was stable and balanced. The number of clean reads obtained per sample was above 97% for Q20 and above 93% for Q30. More than 70% of the clean reads mapped to the chicken reference genome and over 65% map to unique loci. Furthermore, to ensure the accuracy of sequencing results and exclude errors caused by abnormal samples, the pearson product moment correlation coefficient was evaluated among the samples. The biological replicate R^2^≥0.85, the expression pattern similarity between the samples was high, and the RNA-seq results were reliable and could be used for subsequent analyses.

### Analysis of differentially expressed genes between groups

**Figure 2:**
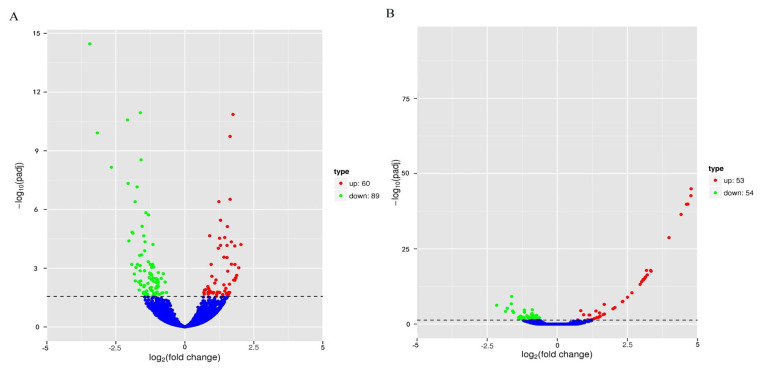
Volcano plot of DEGs among different chicken groups. (A) DEGs between breast muscles of caged hens and free-range hens. (B) DEGs between breast muscles of caged hens and caged cocks; dotted line, fold change (|log_2_FC|)>1. DEGs, differentially expressed genes.

**Figure 3:**
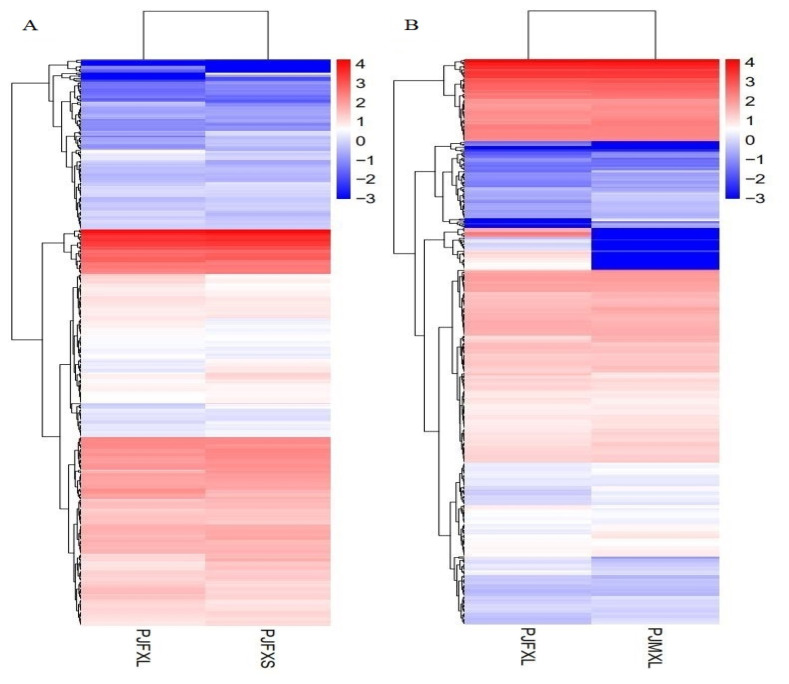
Clustering analysis of DEGs among different chicken groups. DEGs, differentially expressed genes; PJFXL, caged hens group; PJFXS, free-range hens group; PJMXL, caged cocks group; red represents upregulated genes, blue represents downregulated genes; the color transition from blue to red represents log_10_ (FPKM+1) from small to large.

In this study, the expression levels of genes were quantified by FPKM values, and 149 DEGs were identified between the two groups Jingyuan chickens of different feeding patterns; 60 of these genes were upregulated, and 89 genes were downregulated (Figure 2A). There were 107 DEGs identified between the two groups of different sexes of Jingyuan chickens, between the two groups Jingyuan chickens of different sexes, 53 of these genes were upregulated and 54 genes were downregulated (Figure 2B). Clustering analysis of the DEGs helped to visualize the expression levels of these DEGs among the samples, and it highlighted the reproducibility and credibility of the data. The results of the clustering analysis of the DEGs between Jingyuan chickens of different feeding patterns and different sexes groups were good (Figure 3A, B).

### Biological functions of the differentially expressed genes

**Figure 4:**
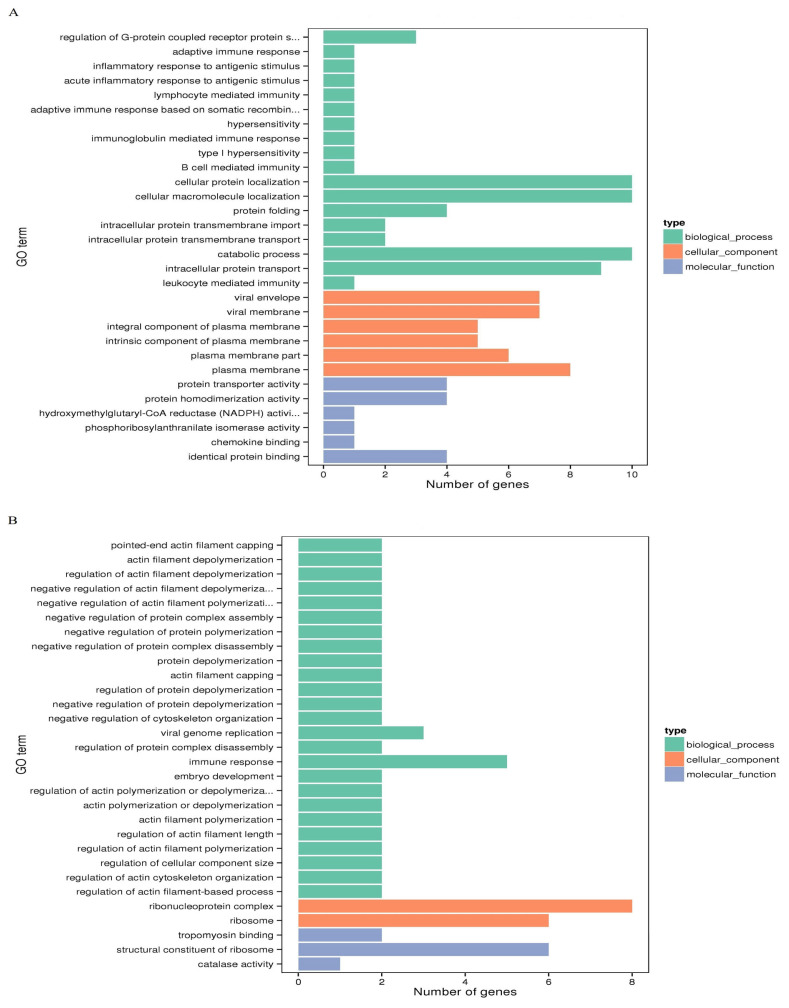
GO functional enrichment analysis of DEGs among different chicken groups. (A) The 30 most significantly enriched GO terms for DEGs of different feeding methods. (B) The 30 most significantly enriched GO terms for DEGs of different sexes. GO, gene ontology; DEGs, differentially expressed genes.

To understand the biological functions involved with the DEGs, GO, and KEGG signaling pathway enrichment analyses were performed. The DEGs among the Jingyuan chickens of different feeding patterns were mainly enriched in 993 GO terms in three major categories, namely biological processes, molecular functions, and cellular components, out of which 105 terms were significantly enriched (p<0.05) (Figure 4A); these included purine nucleoside metabolic process and glutamine family amino acid metabolic process (Table 4). KEGG signaling pathway enrichment analysis results showed that the DEGs were enriched in alanine (Ala), aspartate (Asp), and glutamate (Glu) metabolism as well as purine metabolism. The DEGs among the Jingyuan chickens of different sexes were mainly enriched in 696 GO terms in three major categories, namely biological processes, molecular functions, and cellular components, out of which 83 terms were significantly enriched (p<0.05) (Figure 4B). However, in this case, the purine nucleoside monophosphate metabolic process was not significantly enriched (Table 4), and the KEGG signaling pathway enrichment analysis results showed that the DEGs were not enriched in the IMP-related pathways.

### Protein interaction network analysis of *PDE10A* and *GLUL* genes

**Figure 5:**
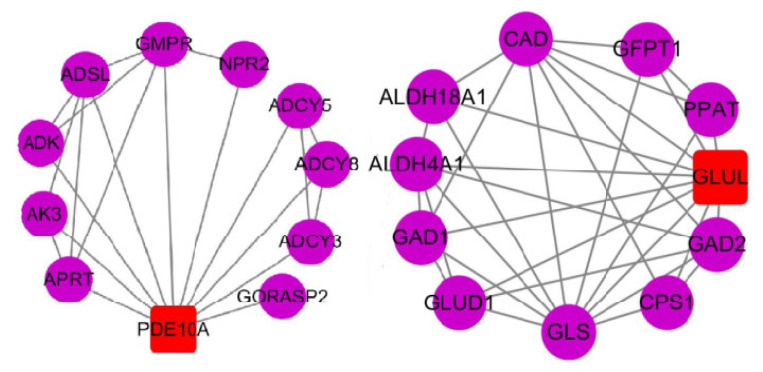
Protein network reciprocity map. The circles show the reciprocal proteins, the squares show the proteins encoded by DEGs, and the connecting lines indicate that the reciprocal relationship between the two still exists at a confidence level of 0.700; DEGs, differentially expressed genes; PDE10A, phosphodiesterase 10A; APRT, adenine phosphoribosyltransferase; AK3, adenylate kinase 3; ADK, adenosine kinase; ADSL, adenylosuccinate lyase; GMPR, guanosine monophosphate reductase; NPR2, natriuretic peptide receptor 2; ADCY5, adenylate cyclase 5; ADCY8, adenylate cyclase 8; ADCY3, adenylate cyclase3; GORASP2, golgi reassembly stacking protein 2; GAD1-2, glutamate decarboxylase 1–2; CPS1, carbamoyl-phosphate synthase 1; GLS, glutaminase; GLUD1, glutamate dehydrogenase 1; ALDH4A1, aldehyde dehydrogenase 4 family member A1; ALDH18A1, aldehyde dehydrogenase 18 family member A1; CAD, carbamoyl-phosphate synthetase 2, aspartate transcarbamylase, and dihydroorotase; GFPT1, glutamine--fructose-6-phosphate transaminase 1; PPAT, phosphoribosyl pyrophosphate amidotransferase.

**Figure 6:**
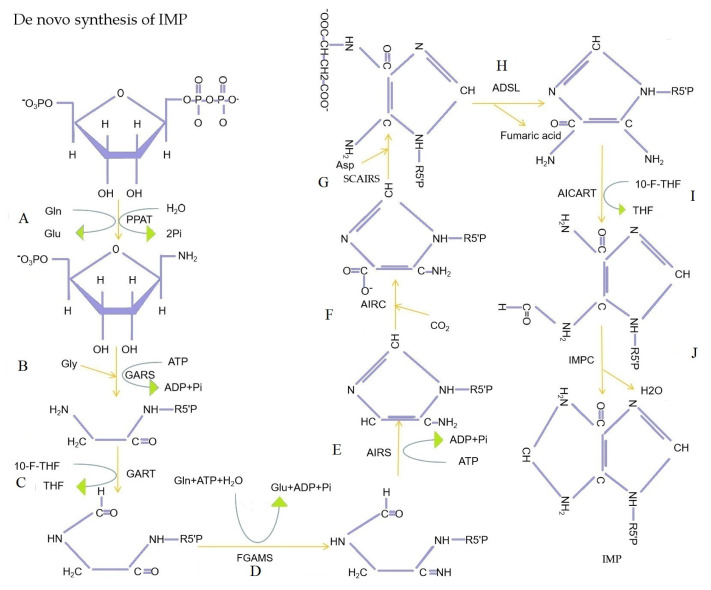
De novo synthesis of IMP. (A) Phosphoribosyl pyrophosphate amidotransferase (PPAT) catalyzes the substitution of pyrophosphoryl acyl group of phosphoribosyl pyrophosphate (PRPP) by the amide nitrogen of Gln. (B) Glycinamide ribonucleotide synthetase (GARS) catalyzed Gly acylation of amino groups of 5-phosphoribosyl amine. (C) Glycinamide ribonucleotide transformylase (GART) catalyzes the transfer of the formyl group of 10-formyltetrahydrofolate to the amino group of glycinamide hydrochloride. (D) the amide of Gln is converted to amidine (R, NH-C=NH) catalyzed by formylglycinamidine ribonuleotide synthetase (FGAMS). (E) Aminoimidazole ribonucleotide synthetase (AIRS) catalyzes closed-loop reactions of formylglycinamidine ribotide. (F) Aminoimidazole ribonucleotide carboxylase (AIRC) catalyzes CO_2_ linkage to the purine ring of aminoimidazole ribonucleotide. (G) Aminoimidazole succinylocarboxamide ribonucleotide synthetase (SCAIRS) catalyzes the condensation of Asp with the carboxyl group of aminoimidazole carboxylic acid ribonucleotides to form amide bond. (H) Aminoimidazole succinylocarboxamide ribonucleotide removal of succinic acid catalyzed by adenylosuccinate lyase (ADSL). (I) Aminoimidazole carboxamide ribonucleotide transformylase (AICART) catalyzes the transfer of the formyl group of 10-formyltetrahydrofolate to the amino group of aminoimidazole aminocarbonyl ribonucleotide. (J) IMP cyclohydrolase (IMPC) catalyzes the closed-loop reaction of 5-formamide-4-imidazole-aminocarbonyl ribonucleotide to form IMP. IMP, inosine monophosphate; ATP, adenosine triphosphate; ADP, adenosine diphosphate; Gln, glutamine; Gly, glycine; Asp, aspartic acid; THF, tetrahydrofolate; 10-F-THF, 10-formyl-tetrahydrofolate.

In transcriptional regulation, protein network regulatory analysis can identify the key candidate genes that potentially regulate important traits. Therefore, we combined the results of GO and KEGG signaling pathway enrichment analyses to further construct protein network interaction analysis maps for the screened key genes *PDE10A* and *GLUL* (Figure 5). The results of the protein network interactions of these two DEGs showed that PDE10A interacted with seven genes in purine metabolism (confidence 0.700), including the *ADSL* gene in the IMP de novo synthesis pathway (Figure 6A-J), while GLUL interacted with eight genes in glutamine metabolism (confidence 0.700), including PPAT in the IMP de novo synthesis pathway. Therefore, the differential deposition of IMP in the breast muscles of Jingyuan chickens with different feeding patterns may be related to the expressions of *GLUL* and *PDE10A* genes.

### PDE10A and GLUL reactome pathway and network cluster analysis

**Figure 7:**
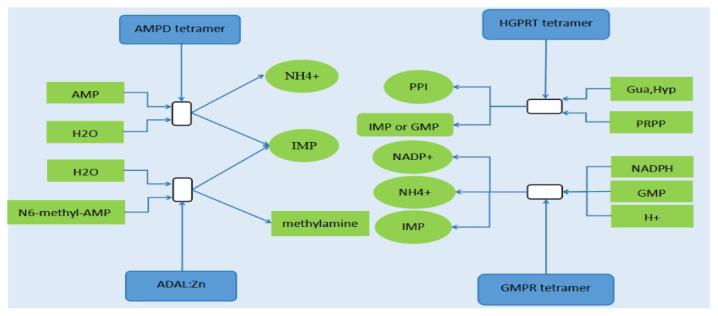
Four reactions of purine salvage involved in the synthesis of IMP. AMPD, adenosine monophosphate deaminase; HGPRT, hypoxanthine-guanine phosphoribosyl transferase; ADAL, adenosine deaminase like; GMPR, guanosine monophosphate reductase; NADPH, nicotinamide adenine dinucleotide phosphate; PRPP, 5-phosphoribosyl 1-pyrophosphate; IMP, inosine monophosphate; AMP, adenosine monophosphate; GMP, guanosine monophosphate; PPI, pyrophosphoric acid; NADP+, oxidized form of nicotinamide adenine dinucleotide phosphate; Gua, guanine; Hyp, hypoxanthine

Reactome pathway analysis showed that the *PDE10A* gene is involved in eight reactome pathways, including the purine salvage. *GLUL* gene is involved in five reactome pathways, including the synthesis, interconversion, and transamination of amino acid as well as the metabolism of amino acids and their derivatives. The purine salvage involves IMP synthesis (Figure 7), while the synthesis and interconversion of amino acids as well as the metabolism of amino acids and their derivatives include the metabolism of Asp, asparagine (Asn), Glu, and glutamine (Gln). Network cluster analysis (confidence 0.900) showed that the *PDE10A* gene is involved in nucleotide metabolism, IMP metabolism, and nicotinate and nicotinamide metabolism (Table 5). On the contrary, the GLUL geng encoded protein is involved in glutamine synthetase, carbon metabolism, amino sugar, and nucleotide sugar metabolism as well as the metabolism and synthesis of many amino acids (Table 6).

### Validation of differentially expressed genes based on quantitative real-time polymerase chain reaction

**Figure 8:**
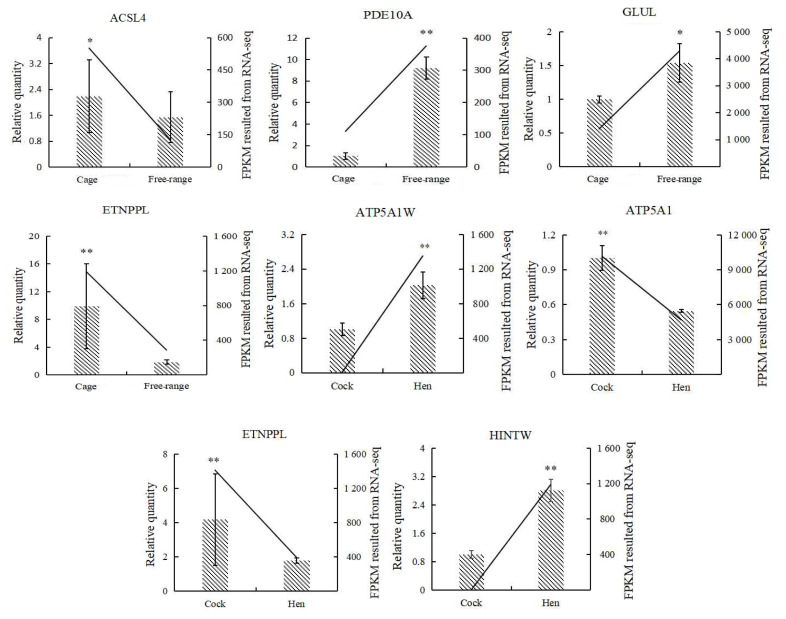
qRT-PCR validation of DEGs (n = 6). DEGs, differentially expressed genes; qRT-PCR, quantitative real time polymerase chain reaction; ACSL4, acyl-CoA synthetase long-chain family member 4; PDE10A, phosphodiesterase 10A; GLUL, glutamate-ammonia ligase; ETNPPL, ethanolamine-phosphate phospholyase; ATP5A1W, ATP synthase F1 subunit alpha W chromosome; ATP5A1, ATP synthase F1 subunit alpha Z chromosome; HINTW, histidine triad nucleotide binding protein W; FPKM, reads per kilobase of exon model per million mapped reads; the straight line is the FPKM value of RNA-seq, histogram is the relative gene expression 2^−ΔΔCt^ value, * p<0.05, ** p<0.01 (one-way analysis of variance).

Analysis of the qRT-PCR validation results for the seven genes showed that the melting curves of all genes were single-peaked, the primers were well-designed, and the validation results were consistent with the trend of RNA-seq results (Figure 8). Hence, the sequencing results were reliable and could be used for subsequent gene function validation.

### Correlation analysis of *PDE10A* and *GLUL* genes expressions with inosine monophosphate content

**Figure 9:**
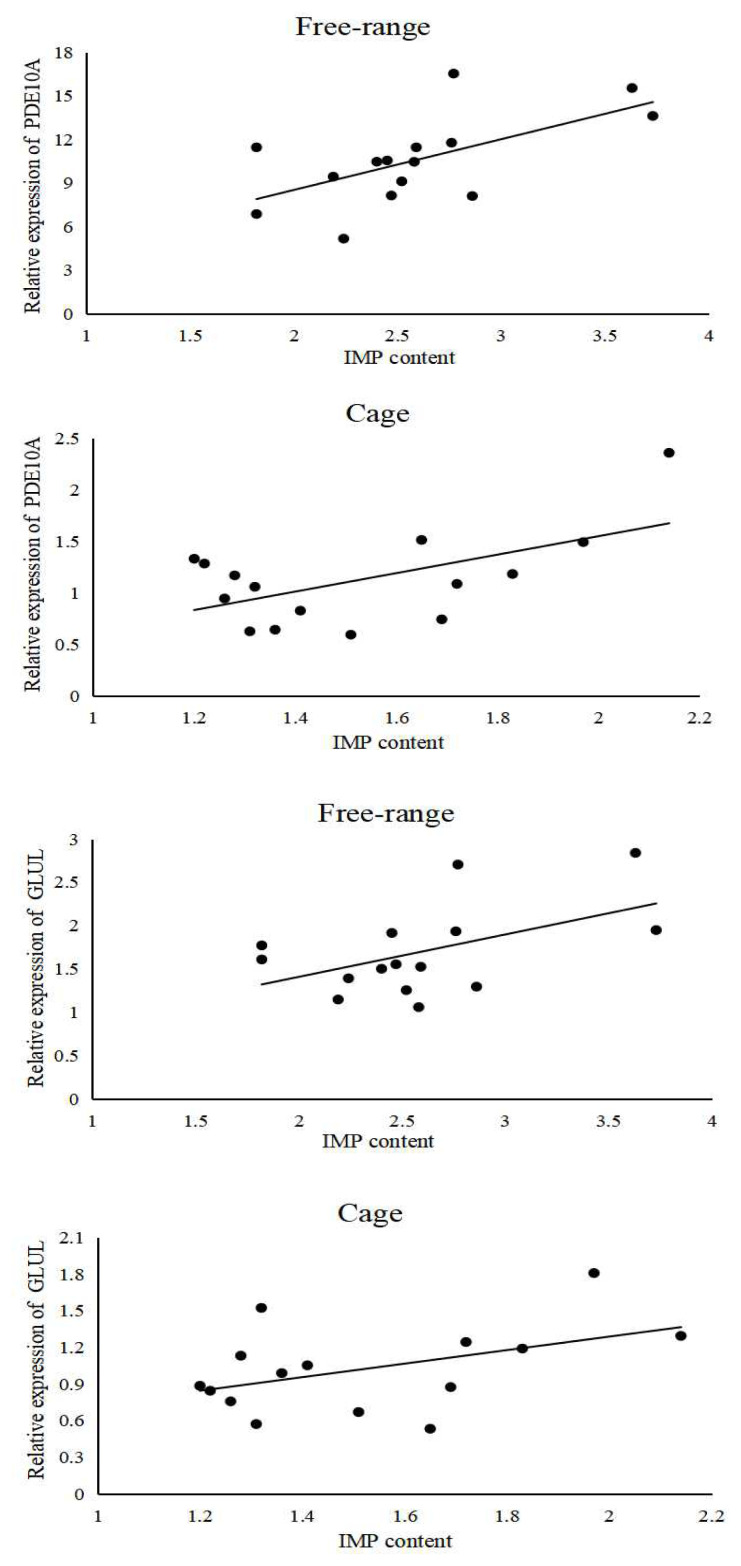
Scatter plot (regression line) of gene expression and IMP content. IMP, inosine monophosphate; PDE10A, phosphodiesterase 10A; GLUL, glutamate-ammonia ligase.

Bivariate correlation analysis between the *PDE10A* gene expression and breast muscle IMP content in free-range chickens showed the correlation coefficient, R^2^ = 0.616 (p<0.05). A similar analysis for caged chickens showed that the correlation coefficient, R^2^ = 0.567 (p<0.05). Similarly, the correlation coefficient, R^2^ = 0.509 (p>0.05) for the correlation between the *GLUL* gene expression and breast muscle IMP content in free-range chickens, and in caged chickens it is R^2^ = 0.44 (p>0.05). The scatter plot (regression line) was drawn to visualize the relationship between the IMP content and gene expression. (Figure 9). *PDE10A* gene expression was significantly and positively correlated with the IMP content in breast muscle tissues (p<0.05), and *GLUL* gene expression was positively correlated with the IMP content in the breast muscle tissues.

## DISCUSSION

IMP is a key factor affecting the quality of meat and an important indicator to evaluate the quality of meat [17]. Therefore, the synthetic degradation of IMP in poultry is of great interest to researchers. Many studies have revealed that IMP content in a poultry is closely related to the breed, feeding pattern, sex, and position, and it is regulated by multiple metabolic pathways and genes [8,11].

The conventional meat quality indicators are shear force, WHC, and cooking loss, and they determine acceptability of the meat. Shear force can visually reflect the muscle tenderness and is used to evaluate the tenderness and juiciness of muscle after steaming [18]. In this study, we have analyzed the shear forces, WHC, and cooking losses as well as the IMP and inosine content in the breast muscle tissues of Jingyuan chickens breast muscle with different feeding patterns and different sexes. Moreover, we have screened the key genes that affect IMP synthesis by RNA-seq. A comparison of the shear forces of the breast muscles of Jingyuan chickens with different feeding patterns and different sexes showed that the breast muscle shear force of caged chickens was significantly higher than that of free-range chickens (p<0.05), and the breast muscle shear force of hens was extremely significantly higher than that of cocks (p<0.01). Therefore, shear force is influenced by both feeding pattern and sex of the chicken, and it is also significantly related to feeding day-age, and breed of the chicken. The lower the shear force of the muscle, within a certain range, the more tender is the meat. The cooking loss for the breast muscles of caged chickens was significantly higher than that of free-range chickens (p<0.05), and the WHC of the breast muscles hens was significantly higher than that of cocks (p<0.05). The results of this study show that the WHC and cooking loss are consistent with the results of previous studies [19], but the shear force results are inconsistent. This inconsistency is probably related to the differences between in the breed and day-age of the chickens, and further population expansion is required for validation.

The breast muscle IMP content in free-range chickens was extremely significantly higher than that of caged chickens (p<0.01), and the breast muscle IMP content in hens was higher than that of cocks, which is consistent with the results of previous studies [8,20]. However, the breast muscle IMP content in Haikang and Pingwu chickens was higher in cocks than that in hens, and some studies have found significant differences in the key enzymes for IMP synthesis in different breeds of chickens. Therefore, the inconsistent results of the studies may be caused by the day-age, breed, and feed nutrition levels [9,21]. A comparison of the inosine content among different chicken groups revealed that the inosine content was extremely significantly higher in caged chickens than that in free-range chickens (p<0.01), while it was only slightly higher in cocks than in hens. Both inosine and IMP belong to the hypoxanthine nucleotides. The IMP is phosphorylated at the 5′-ribose position as compared to inosine; moreover, it is unstable in the meat and can be degraded to inosine and hypoxanthine [22]. Therefore, the negative correlation of inosine with IMP in this study may be related to this phenomenon of IMP degradation. In addition, it was shown that IMP affects muscle tenderness, thereby affecting consumer acceptability of the meat [17,23]. In this study, the IMP content in the breast muscle tissues was significantly negatively correlated (p<0.05) with the breast muscle shear force in free-range hens, indicating that the IMP content affects the tenderness of chicken meat.

To investigate the molecular mechanism of IMP deposition, RNA-seq was performed on breast muscle tissue samples obtained from Jingyuan chickens to screen for the key genes regulating IMP synthesis. These results were combined with bioinformatics to perform functional analysis and regulatory network construction of the screened genes. The IMP de novo synthesis pathway begins with PRPP, one carbon unit, glycine (Gly), Gln, Asp, CO2, and ATP as raw materials, and ten 10 enzymes involved in IMP synthesis [24]. The DEGs that were screened in the Jingyuan chickens of different feeding patterns were found to be involved in glutamine metabolism and purine ribonucleotide metabolism in GO and KEGG signaling pathway enrichment analyses. This may be usefully correlated with the differential deposition of IMP in the breast muscles of caged and free-range chickens; this was confirmed by the correlation analysis of IMP content in the breast muscle tissues with the *PDE10A* and *GLUL* genes. Bioinformatics analysis of the two key genes that were screened *PDE10A* and *GLUL*, revealed that *PDE10A* is directly involved in cyclic adenosine monophosphate (cAMP) biosynthesis, purine salvage and IMP metabolism [25]. In contrast, *GLUL* is the key gene for the synthesis of Gln. The de novo synthesis pathway of IMP starts with the substitution of the pyrophosphoryl acyl group of PRPP by the amide nitrogen of Gln, and this reaction is catalyzed by PPAT [26].

Phosphodiesterase (PDE) catalyzes the breakdown of cyclic adenosine monophosphate (cAMP) and cyclic guanosine monophosphate (cGMP) to adenosine monophosphate (AMP) and guanosine monophosphate (GMP) respectively and regulates their functions [27]. Currently, 21 PDE isozymes encoded by 11 different genes have been identified. The PDE superfamily consists of 11 structurally related but functionally distinct gene families, from *PDE1* to *PDE11*, which have conserved catalytic and regulatory structural domains but differ in their inhibition and excitation patterns, substrate specificity, cellular and tissue distribution characteristics [27,28]. As a member of the PDE superfamily, PDE10A was identified for the first time as a bispecific PDE with cAMP-cGMP activity [27–29]. High PDE10A expression in certain neurons such as in the striatum of the human brain causes various psychiatric/neurodegenerative disorders, including schizophrenia and Huntington’s disease [28,29]. Knock-out and inhibition of PDE10A increases levels of cAMP and cGMP levels in mouse cardiomyocytes and significantly reduces myocardial hypertrophy and myocardial fibrosis caused by chronic neurohormonal stimulation [30]. PDE10A was highly expressed in the breast muscle tissues of free-range chickens, and it showed a significant positive correlation with the breast muscle IMP content. Therefore, it can be inferred that PDE10A may convert AMP to IMP by catabolizing cAMP and cGMP, followed by purine salvage in the presence of AMPD1.

The *GLUL* gene is highly conserved across species, and it is one of the oldest known genes [31]. The GLUL catalyzes the conversion of glutamate and ammonia into Gln in the organism through an enzymatic reaction. This is the sole source of endogenous Gln necessary for several important developmental and metabolic pathways such as cell signaling, cell proliferation, neurotransmitters, nucleotide synthesis, maintenance of acid-base equilibrium, nitrogen metabolism, and ammonia detoxification [32,33]. The *GLUL* gene plays an important role in purine and pyrimidine biosynthesis. In hepatocellular carcinoma cells low concentrations of Gln correlate with purine and pyrimidine biosynthesis and glutamine synthetase activity [34]. Studies showed that in zebrafish hepatocytes, the hippo pathway effector Yap1 induced an increased *GLUL* gene expression and activity that stimulated de novo synthesis of purines and pyrimidines and thus promoted the proliferation of the zebrafish hepatocytes, whereas inhibition of *GLUL* gene expression reduced nucleotide biosynthesis and decreased the proliferation capacity of the hepatocytes [35]. Gln is highly correlated with nucleotides as a prerequisite for purine and pyrimidine synthesis. However, in the present study, there was no significant positive correlation between the *GLUL* gene expression and the breast muscle IMP content in the chickens; this may be related to post-transcriptional regulation or glutamine synthetase activity.

## Figures and Tables

**Table 1:** Primer sequence

Gene symbol	Accession number	Primer sequence (5′–3′)	Annealing temperature (°C)
*^β^-actin*	L08165	F:ATGGACTCTGGTGATGGTGTTAC	60
		R: TCGGCTGTGGTGGTGAAG	
*ACSL4*	XM_004940809.3	F: GCACCGTTGTTATCTTCTG	60
		R: CACATTCATTGAGACCATAGG	
*PDE10A*	XM_015284335.2	F: TGGTCAAGTGGCAAGAAC	60
		R: CTTCCTCGGCTCACAATAG	
*GLUL*	NM_205493.1	F: AGATGGTCATCCGTTTGGC	60
		R: TCACTTCTGCGTTGGTTCCT	
*ETNPPL*	XM_015276580.2	F: CTACAGCAAGGCGGAGAC	60
		R: CCTGAGCACGGACTATCTTC	
*ATP5A1W*	XM_429118.6	F: GAACTGGCACTGCTGAGGT	60
		R: CGGGCAATACCATCACCAA	
*ATP5A1*	AF332870.1	F: GAAACGCAGGCTGGTGATGT	60
		R: AGCAGAACCTACACGGGACA	
*HINTW*	NM_204688.2	F: GTTGTGGACGAGGAGTGCC	60
		R: ACGCCCAAGAAGAGGTGC	

*β-actin*, bate-actin; *ACSL4*, acyl-CoA synthetase long-chain family member 4; *PDE10A*, phosphodiesterase 10A; *GLUL*, glutamate-ammonia ligase; *ETNPPL*, ethanolamine-phosphate phospho-lyase; *ATP5A1W*, ATP synthase F1 subunit alpha W chromosome; *ATP5A1*, ATP synthase F1 subunit alpha Z chromosome; *HINTW*, histidine triad nucleotide binding protein W.

**Table 2:** Conventional meat quality index of the breast muscle of Jingyuan chickens

Feeding pattern	Sex	Shear force (kg)	WHC (%)	Cooking loss (%)
Caged	Hen	15.640±3.867^a^	0.435±0.046	0.239±0.043^A^
Free-range		13.272±3.574^b^	0.432±0.054	0.181±0.034^B^
Caged	Cock	12.083±2.241^A^	0.400±0.030^a^	0.213±0.060
	Hen	15.640±3.867^B^	0.435±0.046^b^	0.239±0.043

WHC, water holding capacity.

a,b Means within same column with different superscript letters are different (p<0.05).

A,B Means within same column with different superscript letters are different (p<0.01).

**Table 3:** IMP and inosine content of the breast muscle of Jingyuan chickens

Feeding pattern	Sex	IMP content (g/kg)	Inosine content (g/kg)
Caged	Hen	1.525±0.294^A^	0.327±0.067^A^
Free-range		2.589±0.538^B^	0.196±0.089^B^
Caged	Cock	1.484±0.298	0.359±0.079
	Hen	1.525±0.294	0.327±0.067

IMP, inosine monophosphate.

A,B Means within same column with different superscript letters are different (p<0.01).

**Table 4:** Results of RNA-seq quality control data analysis

Sample name^1)^	Clean reads	Q20^2)^ (%)	Q30^3)^ (%)	GC content (%)	Total mapped (%)	Multiple mapped (%)	Uniquely mapped (%)
PJFXL	43 420 860	97.44	93.47	56.35	70.92	2.62	68.3
	48 037 058	97.36	93.26	56.72	70.62	2.17	68.46
	53 633 184	97.34	93.18	56.26	70.02	3.04	66.99
PJMXL	48 122 968	97.48	93.51	55.92	71.63	2.47	69.16
	38 940 706	97.38	93.32	55.97	72.14	2.49	69.65
	51 570 778	97.64	93.91	55.77	71.8	2.81	68.99
PJFXS	49 682 728	97.5	93.6	56.12	70.35	2.96	67.39
	49 672 972	97.34	93.21	56.65	70.01	4.02	65.99
	48 728 926	97.53	93.66	55.86	70.93	2.97	67.96

1) PJFXL, caged hens group; PJMXL, caged cocks group; PJFXS, free-range hens group.

2) The Q value refers to the base calling process during sequencing, and it gives the probability of error for the identified bases; Q20, the mass value is Q20, then the probability of misidentification is 1%.

3) Q30, the mass value is Q30, then the probability of misidentification is 0.1%.

**Table 5:** Summary of GO terms and KEGG pathway enrichment of DEGs (IMP-related)

Items	GO ID/KEGG ID	Description	p-value	Genes^1)^
Caged vs free- range	GO:0042278	Purine nucleoside metabolic process	0.0158	*HMGCR, PDE10A, MAT1A, AKAP7*
	GO:0009064	Glutamine family amino acid metabolic process	0.0215	*GLUL ENSGALG00000021609*
Hen vs cock	GO:0009126	Purine nucleoside monophosphate metabolic process	0.1836	*ATP5A1W, ATP5A1*
Caged vs free- range	gga:396489	Alanine, aspartate and glutamate metabolism	0.0446	*GLUL, DDO, GADL1*
	gga:421574	Purine metabolism	0.0835	*PDE10A, ADA*

GO, gene ontolog; KEGG, Kyoto encyclopedia of genes and genomes database; DEGs, differentially expressed genes; IMP, inosine monophosphate.

1) *HMGCR*, 3-hydroxy-3-methylglutaryl-CoA reductase; *PDE10A*, phosphodiesterase 10A; *MAT1A*, methionine adenosyltransferase 1A; *AKAP7*, A-kinase anchoring protein 7; *GLUL*, glutamate-ammonia ligase; *ENSGALG00000021609*, consult http://asia.ensembl.org/website; *ATP5A1W*, ATP synthase F1 subunit alpha W chromosome; *ATP5A1*, ATP synthase F1 subunit alpha Z chromosome; *DDO*, D-aspartate oxidase; *GADL1*, glutamate decarboxylase like 1; *ADA*, adenosine deaminase.

**Table 6:** PDE10A and GLUL network cluster analysis

Gene	Cluster	Description	FDR
*PDE10A*	CL:18025	Purine metabolism, nicotinate and nicotinamide metabolism	1.26×10^−9^
	CL:18088	Mixed, incl. nucleotide salvage and nucleotide metabolism	5.84×10^−5^
	CL:18028	Metabolism of nucleotides and IMP metabolic process	4.09×10^−8^
*GLUL*	CL:17467	Glutamine synthetase, beta-grasp domain and glutaminase	9.48×10^−8^
	CL:17443	Arginine biosynthesis and proline catabolism	7.66×10^−14^
	CL:6404	Mixed, incl. GABA synthesis and vitamin D	5.83×10^−5^
	CL:17456	Proline catabolism and pyrroline-5-carboxylate reductase dimerisation	5.83×10^−5^
	CL:17106	Carbon metabolism, amino sugar and nucleotide sugar metabolism	8.80×10^−3^

*PDE10A*, phosphodiesterase 10A; *GLUL*, glutamate-ammonia ligase; FDR, false discovery rate; IMP, inosine monophosphate; GABA, *γ*-aminobutyric acid.
